# The Effect of Passive and Active Education Methods Applied in Repetition Activities on the Retention of Anatomical Knowledge

**DOI:** 10.1002/ase.1924

**Published:** 2019-11-06

**Authors:** Jan G.M. Kooloos, Esther M. Bergman, Marieke A.G.P. Scheffers, Annelieke N. Schepens‐Franke, Marc A.T.M. Vorstenbosch

**Affiliations:** ^1^ Department of Anatomy Radboud University Medical Center Nijmegen The Netherlands; ^2^ Zuyderland Academy Zuyderland Medical Center Heerlen The Netherlands

**Keywords:** gross anatomy education, medical education, biomedical education, undergraduate education, knowledge retention, active learning, repetition

## Abstract

This study examines the long‐term retention of anatomical knowledge from 180 students after various repetition activities. The retention of anatomical knowledge was assessed by multiple‐choice tests at five different points in time: before and after a course in Functional Anatomy, before and after repetition activities that occurred 14 weeks after this course, and 28 weeks after this course to establish long‐term retention. Students were divided into five groups: one without any repetition activity, one with a restricted repetition activity (the multiple‐choice test), and three groups that were offered repetition activities (traditional lecture, e‐learning module, and small group work in the dissection room). During all three repetition activities the same information was conveyed, and this content was not revisited in other courses for the duration of the study. The results showed that students who did not engage in a repetition activity scored significantly lower on the long‐term retention test compared to all other groups (ANCOVA: *P* = 0.0001). Pair‐wise comparison with estimated means showed that the other four groups, regardless of the type of repeating activity, did not differ in the amount of knowledge they retained during any of the five assessments (*P* = 0.008, *P* = 0.0001, *P* = 0.001, and *P* = 0.0001, respectively). This study suggests that the type of repetition activity has no effect on knowledge retention both immediately following the activity and in the long term. It is concluded that the repetition of anatomical knowledge in any form is beneficial for students and will likely improve student outcomes in a curriculum that builds on prior knowledge.

## Introduction

The aim of medical education is to achieve long‐term learning among students, resulting in a cohesive framework of knowledge, ready to be applied in the clinic. However, forgetting is a natural feature of memory: if knowledge is not used, it is most likely unimportant and can therefore be erased (Kerfoot and Brotschi, 2009; Hardt et al., 2013). In this study, the role of repetition in students’ long‐term knowledge retention will be further explored.

The forgetting of knowledge is a negatively accelerated function, better known as “Ebbinghaus’ forgetting curve” (Ebbinghaus, 1885). This curve typically shows a rapid retention loss during the first retention intervals, after which the loss decreases and eventually stabilizes (Ebbinghaus, 1885; Roediger and Karpicke, 2006; Custers, 2010). Although Ebbinghaus’ conclusions are based on nonsense words, which are words that cannot be connected to existing knowledge, more recent research has shown that the same curve applies for meaningful knowledge retention as well (Conway et al., 1992; Bacon and Stewart, 2006; Custers, 2010). In most laboratory research on knowledge retention and the forgetting curve, retention intervals of hours or days are used (e.g., Roediger and Karpicke, 2006). In medical curricula, however, intervals of months or sometimes even years are present between the initial learning of knowledge and the moment this knowledge is expanded in a subsequent course or needs to be applied during clinical rotations. Experiments conducted with such long intervals show the same retention curve: knowledge retention decreases sharply during the first interval, and then the decline stabilizes (Conway et al., 1992; Semb and Ellis, 1994; Cepeda et al., 2008; Custers, 2010; Kooloos et al., 2012). In his review study on long‐term retention of basic sciences knowledge, Custers (2010) concludes that irrespective of the subject matter or type of knowledge or skill, after a retention interval of one year, about 33% of the gained knowledge is lost. After two years, this loss increases to about 50%. Although many factors, such as students’ individual learning abilities or retrieval conditions, might contribute to the longevity of retention, one element seems to be the most important: repetition.

Knowledge retention is improved by one or more encounters with the same previously learned knowledge (Custers, 2010). “Spaced education,” in which shorter educational encounters are spaced and repeated over time, results in more efficient learning and improved retention compared with “bolus education,” a single but elaborate educational encounter in a short amount of time (Kerfoot et al., 2007). Bolus education often motivates students to study by cramming, which may lead to sufficient short‐term results, but it ultimately will have an adverse effect on the long‐term retention of knowledge and skills (Deslauriers and Wieman, 2011; Lindsey et al., 2014). Each revisit slows knowledge loss, and thus repetition improves permanent knowledge retention. Medical schools have tried to facilitate revisits to earlier learned knowledge through so‐called spiral curricula (Harden and Stamper, 1999). In a spiral curriculum, the teaching of topics is spaced over multiple months or years, with each successive encounter building on the previous one and thus deepening the associated knowledge. Similarly, teachers stimulate students to reach higher levels of professional competencies using so‐called scaffolding strategies (Fernández et al., 2001; Sanders and Welk, 2005). When using scaffolding strategies, learning can only be successful when the foundation is strong and solid so that each new element can rest firmly on the ones below (Paechter, 2004). Revisiting previously learned knowledge reinforces the students’ foundation of knowledge, providing a solid base for further knowledge expansion. But in practice, teachers tend to continue education where they had left off months or years before and may be surprised and/or annoyed by students who have forgotten the corresponding knowledge. Although repetition of previously taught topics is described as a key feature of spiral curricula (Harden and Stamper, 1999), educational programs rarely provide “systematic revisitation” of previously learned knowledge (Lindsey et al., 2014). However, before implementing repetition in curricula, the following question needs to be addressed: which learning method is most successful when repeating previously taught topics?

It is not self‐evident which learning method should be used for repetition. Current literature suggests that, at least during initial learning, active learning methods outperform passive ones because of improved student engagement, self‐esteem, and attitude (Johnson et al., 1998; Norman and Schmidt, 2000; Michael, 2006; Gleason et al., 2011; Minhas et al., 2012; Markant et al., 2016). Active learning methods contribute to higher academic achievement in terms of summative test scores, although not overwhelmingly, due to relatively small effect sizes (Prince, 2004), and are therefore under ongoing investigation. A recent review of 225 studies in the field of undergraduate science curricula concludes that there is a general improvement of 6% on examinations when students are taught by active learning methods (Freeman et al., 2014). Hora (2014) challenged Freeman and colleagues’ interpretation, which is based on rigid classification of studies into either passive or active learning methods, suggesting instead a more fine‐grained approach to categorizing studies. Nevertheless, most studies that are included in Freeman and colleagues’ analyses suggest that active learning methods are beneficial for initial knowledge uptake (Freeman et al., 2014). Yet, it remains unknown if this same relationship exists for the repetition of previously learned knowledge.

The idea that active learning methods outperform passive ones in the storage of information during the initial learning stage and as a consequence in the retrieval of that same information stems also from the theory of memory. There are three core processes of memory: encoding, storage, and retrieval of information. New information is encoded by the sensory system into the working memory and stored in the long‐term memory in schemas that are built from the new information as well as information that was already stored in existing schemas (e.g., van Merriënboer and Sweller, 2010). The process of encoding, storing, and retrieving is enhanced by emotional arousal (Crowley et al., 2019). Arousal will help to construct stronger and larger schemas during initial learning, which makes it easier to retrieve the learned information from long‐term memory (van Kesteren et al., 2012). Active learning methods try to arouse the learner by giving them the opportunity to control the information that is experienced (Markant et al., 2016). In contrast, when new information is taught with a passive learning method, this information is stored with less connections to the existing schemas, and hence retrieval becomes more cumbersome. There are many ways to put the learner in control, for example, goal‐driven exploration, physical interaction, self‐pacing, meta‐cognitive monitoring, and social interaction (Markant et al., 2016). The taking of a test is also an active learning method. Moreover, taking tests gives better results in the recall of the initially learned knowledge than restudying the same material, at least after a time‐span of about a week. This so‐called testing effect (Roediger and Karpicke, 2006) is studied in different ways (Binks, 2018), but not yet in connection to an existing curriculum and with a timespan of several months.

The aim of this study is to explore whether the supposed benefits of active learning methods in the initial learning stage also apply when previously learned knowledge is revisited. Based on the theory of memory described above, it was expected that students who were offered more active learning methods when revisiting earlier learned knowledge would outperform students who were offered a more passive learning method in the recall of anatomical knowledge that was initially learned 14 weeks earlier. In the same line of thought, it was expected that students who had revisited anatomical knowledge with active learning methods would recall more knowledge in a long‐term retention test another 14 weeks later than students who had revisited the same anatomical knowledge with a passive learning method.

To make these hypotheses operational, it is necessary to define active and passive learning methods in more detail. Learning methods are regarded as active when components that are crucial to arousal and activation are met: are students purposely actively involved? Is student engagement encouraged? Is cooperation, collaboration, or even competition between the students promoted? Is the autonomy of students addressed (Prince, 2004; Michael, 2006)? In the present study, the passive learning method is defined as a method involving none of the components described above, that is, the traditional lecture. This method does not suggest that students become completely passive. Some activity of the students is required: they need to mentally process the material that is fed to them by the teacher during a traditional lecture. Two active learning methods that are supposed to increase engagement and thereby result in a larger learning gain are also used in this study. One method invites students to get involved with the study material by means of an e‐learning module with educational questions. With the other active learning method, engagement is assumed to be larger than with the e‐learning module because a meaningful context, in terms of a location (the dissection room) and study material (relevant anatomical specimens), is added.

## Materials and Methods

### Setting

This study was conducted from October 2013 to May 2014 at the Radboud University Nijmegen Medical Center in Nijmegen, The Netherlands. Students in the Netherlands usually enter university immediately after finishing high school when they are around 19 years of age. In Nijmegen, the medical and biomedical students are mixed during their first year in their basic science courses. In their second year, the two curricula diverge. Medical students are offered more clinically directed courses, while biomedical students are offered more research‐oriented courses.

At the time of the study, the first‐year bachelor curriculum in Nijmegen consisted of 10 consecutive basic sciences and systems courses, such as Functional Anatomy, Biochemistry and Biophysics, Circulation and Respiration, and Movement and Motor Control. These 10 first‐year courses all lasted four weeks and ended with a summative examination. This study explored knowledge retention of material covered in the Functional Anatomy course, which was the second course in the first year (October 2013) that covers basic anatomical, radiological, and some physiological knowledge of the thorax, abdomen, and pelvis. In this course, like all courses in the first year, self‐study assignments, small group work, practical sessions, and interactive lectures were offered to the students. In the Functional Anatomy course in particular, 21 assignments for self‐study took up about 65% of the time. The remaining 55 hours of contact time were divided as follows: seven traditional lectures; six practical sessions in the dissection room to study prosections; three e‐learning modules on the thorax, abdomen, and pelvis; seven small group sessions; eight interactive lectures; and one body‐painting session. This study ended during the Movement and Motor Control course, which was the ninth first‐year course (May 2014).

### Participants and Ethics

This study was performed among roughly 450 first‐year medical and biomedical students, 288 of whom were included in this study. After completion of the study 7.5 months later, the long‐term test results of 180 students could be collected. Of these, 54 students (30%) were male and 126 students (70%) were female, which corresponds to the male/female ratio of the (bio)medical student population. Their average age was 18.8 ± 1.3 years. Participation was voluntary and informed consent was obtained from each participant.

Medical education research in the Netherlands is exempt from ethical approval. Nevertheless, this study was approved by the Department of Evaluation, Quality, and Development at the Institute of Scientific Education and Training of the Radboud University Medical Center Nijmegen, Nijmegen, The Netherlands. The students were offered a small financial reward (EUR 25,00) for their participation and a meal during the repetition activities.

### Procedure and Constitution of the Groups

**Figure 1:**
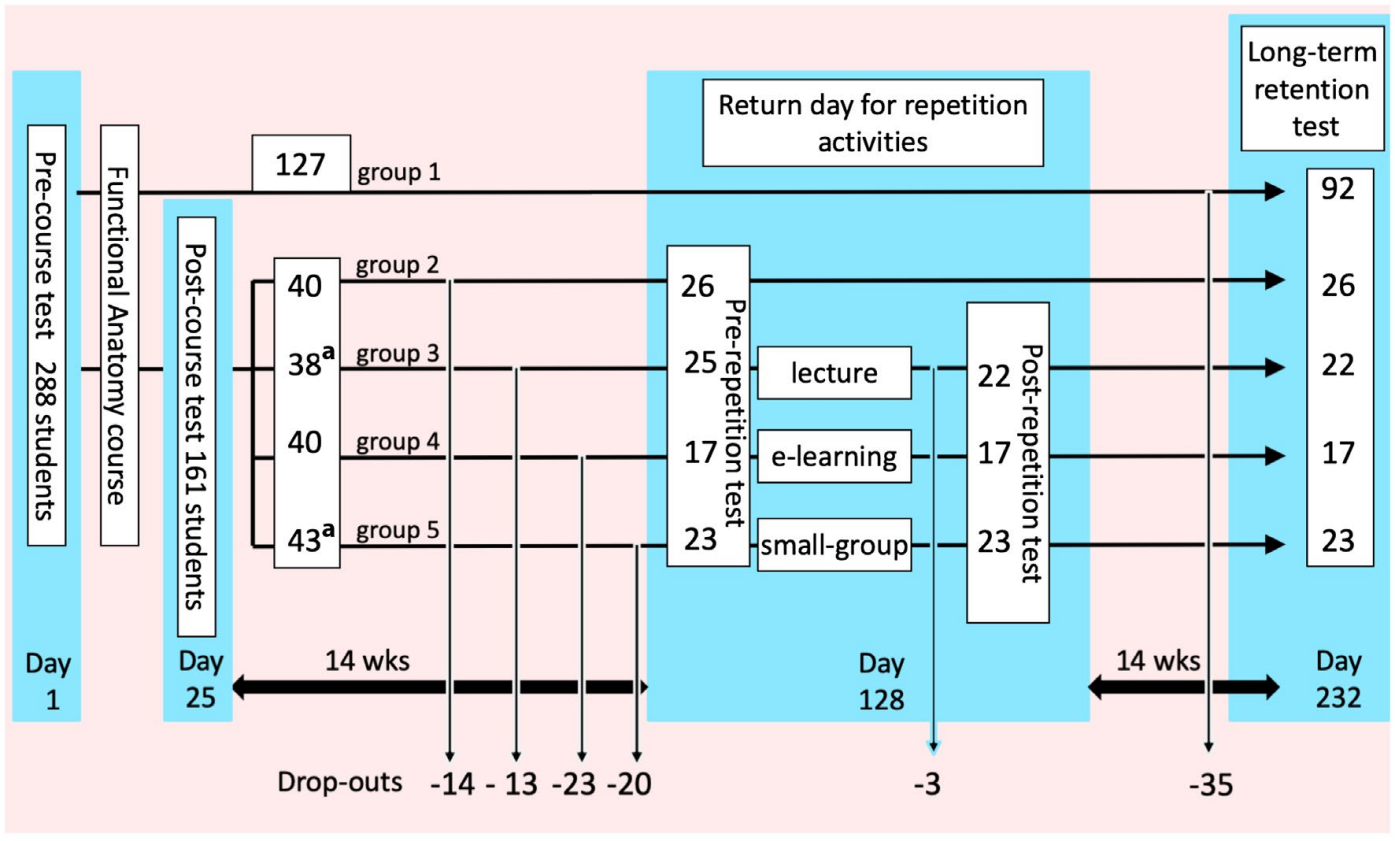
Numbers of students in the different stages of the study. After the pre‐course test and the regular first‐year course, students who took the long‐term retention test were placed in Group 1. The students that took the post‐course test were denominated to Groups 2, 3, 4, or 5. Participants in Group 2 did not receive any repetition activity, but took the pre‐repetition test on the return day. Participants in Groups 3, 4, and 5 took a pre‐repetition test, participated in different repetition activities, rehearsed a selection of topics learned in the regular course, and took a post‐repetition test. At the bottom of the figure, the numbers of students that were lost in between the different tests are depicted. ^a^Two students who were in Group 3 accidentally took the classes of Group 5 on the return day.

The number of students during the different stages of the study is displayed in Fig. 1. On Day 1, all first‐year students who came to the university to take regular classes at the start of the Functional Anatomy course were invited to complete the pre‐course test. The purpose of the pre‐course test was to establish the entrance knowledge level for this study. One day before the end of the course (Day 25), all students attending the regular, yet optional classes were invited to take the post‐course test. The post‐course test was administered to establish the knowledge gained as a result of the learning activities that took place during the Functional Anatomy course. The post‐course test was separate from the summative test associated with the first‐year course (Day 26). A substantial number of students who took the pre‐course test did not show up for class activities on the day of the unannounced post‐course test, but a large number of these students were willing to take the long‐term retention test six months later. These 127 students were assigned to Group 1. The students who took both the pre‐course and post‐course test were equally distributed into Groups 2–5 based on gender and age. All students in Groups 2–5 were asked to gather at the university on Day 128 (the return day) for repetition activities. During the return day, all students took a pre‐repetition test to assess any potential knowledge loss that occurred during the 14 weeks’ time between the end of the Functional Anatomy course and the return day. Students in Group 2 took the pre‐repetition test without further activities. This group was added to find out the effect of the pre‐repetition test itself on the retention test. Students in Groups 3, 4, and 5 participated in different repetition activities in which a selection of the topics encountered in the regular course was revisited. Afterward, they took the post‐repetition test in order to establish the differences in the amount of knowledge gain among Groups 3, 4, and 5. Students of all groups were invited to take a final long‐term retention test after a second period of 14 weeks. This invitation took place during an obligatory small group educational activity in the first‐year course in May. All students who took this obligatory small group activity were invited to take the long‐term retention test.

### Taking the Tests

The pre‐course test, post‐course test, pre‐repetition test, post‐repetition test, and retention test all consisted of the same 40 multiple‐choice questions, but in a different sequence each of the five times the test was taken. Questions contained an average of three answer choices with a range of 2–4 answer choices. Students were not informed of their test performance, since feedback could have impacted knowledge retention. Some examples of test questions are included in the Supplementary Material Appendix File.

The maximal duration of the study was limited to 7.5 months, due to the review of a large part of the material from the Functional Anatomy course in subsequent first‐year courses. Given this timeframe and the length of the course itself (26 days), the maximal amount of time that could be allotted between the course and the completion of the study was roughly 28 weeks. Therefore, 14 weeks were allotted between the post‐course test and the repetition activities, and an additional 14 weeks were allotted between the repetition activities and the final long‐term retention test (see Fig. 1). The majority of topics initially learned in the Functional Anatomy course were re‐visited in subsequent coursework prior to completion of the study. For instance, the heart, lungs, and kidneys were reviewed prior to the long‐term retention test. Therefore, questions about these organs were excluded from the study. In other words, the long‐term retention test consisted only of questions that were not re‐visited in the 28 weeks of this study (viz. body wall, inguinal canal, pelvic floor, pelvic organs, and embryology).

### Repetition Activities

Groups 2–5 were invited to take tests and do repetition activities on the return day (Day 128). Students were informed to which repetition activity they were allotted shortly before the return day. The repetition activities for Groups 3, 4, and 5 were a traditional lecture, an e‐learning module, and small group work in the dissection room, respectively. As stated in the introduction, the traditional lecture was chosen (for Group 3) to represent the passive learning method, since it involves listening to the teacher and therefore requires little effort from the students themselves. During the lecture, the students were not allowed to ask the teacher any questions. This was done to avoid student‐teacher interaction, which might stimulate uptake of knowledge. An e‐learning module was chosen (for Group 4) to represent the middle ground between passive and active learning methods. During this session, students individually read the subject material and answered associated practice questions on a computer. Students were allowed to use their study books to answer the questions and were given feedback on their performance, which was embedded in the e‐learning module. However, students had to work independently, that is, without collaborating with other students, and were not allowed to ask the teacher any questions. A structured small group learning session in the dissection room (Kooloos et al., 2012) was chosen (for Group 5) to represent the most active learning method, as students had to collaborate and discuss the information among themselves and with the teachers and were invited in this way to get really involved with the study material. Care was taken to present exactly the same study material (figures or texts) during all three types of repetition activities. However, the students attending the small group learning session also had anatomical preparations (prosections) at their disposal. All three types of repetition activities took place on the same day and at the same time, were designed to last 1.5 hours, and were scheduled outside of regular class times.

### Statistics

To determine whether there is a difference in the long‐term retention test scores among the five groups of students when controlling for the pre‐course test, an ANCOVA was performed with the long‐term retention test scores as the dependent variable, the group as the fixed variable, and the pre‐course test as a covariate. When significant differences were found, pair‐wise comparisons based on estimated marginal means were used to elucidate differences among the five groups. Partial eta‐squared was used for the effect size. To evaluate whether knowledge retention differs across the five tests among the three groups that engaged in repetition activities, a repeated measures ANOVA for both within‐subjects and between‐subjects was performed. Cronbach’s alpha was used to assess the reliability. According to DeVellis (2017), a reliability of 0.5–0.6 is regarded as poor, 0.6–0.7 as moderate, and 0.7 or higher as acceptable to good. Kendall tau‐B was used to estimate the convergent validity of the five anatomical knowledge tests. Kendall tau‐B is an ordinal measure of correlation and was used to indicate the concordance of the order (ranks) of the score of the participants in the different tests. According to Taylor (1990) correlation coefficients can be interpreted as follows: below 0.35 indicates a weak correlation, between 0.35 and 0.67 a moderate, and above 0.67 a strong correlation. All statistical tests were performed using SPSS statistical package, version 25.0.0.1 (IBM Corp., Armonk NY).

## Results

### Numbers of Students

From the larger cohort of roughly 450 students, 288 completed the pre‐course test (see Fig. 1), from which 161 also completed the post‐course test. The 127 students who did not take the post‐course test were not actively avoiding the post‐course test. They merely did not show up at scheduled voluntary class activities during which the post‐course test was given, namely without advanced notice. From these 127 students, 92 were willing to take the long‐term retention test six months later. Those 161 students who did complete the post‐course test were distributed into Groups 2–5. Their group sizes were 40, 40, 40, and 41, respectively. From these, 70 students dropped out (14, 13, 23, and 20, respectively), and 91 students were present on the return Day 14 weeks later. Two students from Group 3 joined the repetition activity of Group 5 accidentally, and three students from Group 3 left without taking the post‐repetition test, resulting in final sample sizes of Groups 2–5 being 26, 22, 17, and 23, respectively.

After the second period of 14 weeks, the long‐term retention test was offered to all students. Attendance for this retention test was maximized because this test was offered during an obligatory in‐class work session of the first‐year course that was scheduled at that time.

### Tests and Test Scores

The scores of the students in the different groups are depicted in Table 1. The students scored about 37% on the anatomical knowledge test at the start of the first‐year course on Functional Anatomy, and their scores increased to about 68% at the end of this course. Fourteen weeks later, scores declined to about 51%. After the repetition activities, the scores on the anatomical knowledge test raised again to about 73% and fell down to about 55% for the final long‐term retention test, except for Group 1 who scored about 47% in the long‐term retention test. Assuming that Group 1 would have had similar post‐course test scores (about 68%) as the other groups if they had taken this test, Group 1 lost about 30% (21 percentage points) of their anatomical knowledge between the end of the course and the end of the study. On the other hand, Groups 2–5 lost an average of about 20% (13 percentage points).

**Table 1:** Average Scores of Students per Group on Each Test

Group	N	Pre‐course test mean (±SD)	Post‐course test mean (±SD)	Activities during the return day			Long‐term retention test mean (±SD)	*P*‐values (ANCOVA)
				Pre‐repetition test mean (±SD)	Repetition activity mean (±SD)	Post‐repetition test mean (±SD)		
1	92	37.0 (±7.0)	–	–	–	–	47.3 (±9.3)	n.a.
2	26	37.5 (±5.8)	69.3 (±10.8)	52.8 (±10.5)	–	–	53.0 (±9.3)	0.008
3	22	37.3 (±10.3)	66.5 (±10.3)	50.0 (±11.8)	lecture	73.0 (±9.8)	55.5 (±10.5)	0.0001
4	17	36.5 (±7.8)	69.0 (±10.8)	50.5 (±8.0)	e‐learning	72.5 (±10.3)	55.5 (±9.8)	0.001
5	23	36.5 (±7.3)	65.8 (±11.8)	51.8 (±10.0)	small group	73.5 (±12.8)	55.5 (±10.8)	0.0001

Average scores are expressed in means (± SD) in percentages. The time between the post‐course test and the return day was 14 weeks. The time between the return day and the long‐term retention test was also 14 weeks. Regarding the last column, the significance levels for Group 1 < Groups 2, 3, 4, and 5 were calculated with pair‐wise comparisons based on estimated means.

The pre‐course test showed a particularly low reliability (Cronbach’s alpha −0,022; see Table 2), indicating a highly random measure. Considering the tests consisted of only 40 items, the other alphas were reasonably satisfactory. The post‐course test and the post‐repetition test showed alphas of 0.634 and 0.652, respectively, indicating moderate reliability. The pre‐repetition test and the long‐term retention test had smaller alphas of 0.456 and 0.505, respectively, indicating poor reliability.

**Table 2:** Statistical Analysis of the Five Tests Used in This Study

Test data	Pre‐course test	Post‐course test	Activities during the return day		Long‐term retention test
			Pre‐repetition test	Post‐repetition test	
MC‐questions (N)	40	40	40	40	40
Participants (N)	180	88	88	62	180
Cronbach’s alpha^a^	−0.022	0.634	0.456	0.652	0.505
Score, mean (±SD)	14.79 (±2.94)	26.99 (±4.31)	20.53 (±4.07)	29.21 (±4.33)	20.39 (±4.10)
Score%, mean (±SD)	40.97 (±7.35)	67.47 (±10.77)	51.32 (±10.17)	73.02 (±10.82)	50.97 (±10.25)

Note

that all tests consisted of the same 40 questions, but in a different order.

a Cronbach’s alpha 0.5–0.6 = poor reliability; 0.6–0.7 = moderate; 0.7–0.8 = acceptable; 0.8–0.9 = good. The time between the post‐course test and the return day was 14 weeks. The time between the return day and the long‐term retention test was also 14 weeks; MC‐questions: multiple‐choice questions.

Kendall tau‐B’s correlation coefficient of the long‐term retention test in relation to the pre‐course test, the post‐course test, the pre‐repetition test, and the post‐repetition test were 0.133, 0.431, 0.329, and 0.453, respectively. All these correlations were significant (*P* < 0.01). So, at least the post‐course test and the post‐repetition test correlated moderate to the long‐term retention test.

The ANCOVA showed a significant difference in long‐term retention among the five groups (df = 180; F = 7.325; *P* = 0.0001). The pair‐wise comparisons based on estimated means showed that scores on the long‐term retention of Group 1 were lower than the scores of all the other groups; for Groups 2–5, *P* = 0.008, *P* = 0.0001, *P* = 0.001, and *P* = 0.0001, respectively (see Table 1). The pair‐wise comparisons based on estimated means also showed that there were no differences among the scores of Groups 2, 3, 4, and 5. These results indicate that the test‐only group as well as the lecture group, the e‐learning group, and the collaborative learning session group all performed equally well and performed better than Group 1 in the retention test.

Partial eta‐squared indicated a moderate effect size of 0.144. Repeated measures ANOVA for within‐subject effects on Groups 3, 4, and 5 showed a significant difference (df = 1; F = 174.1; *P* = 0.001), which means that the students’ scores differed on all five tests. Repeated measures ANOVA for between‐subjects effects showed no differences among the scores of Group 3, 4, and 5 (df = 2; F = 0.006; *P* = 0.994). This result indicates that the scores of the students in Groups 3, 4, and 5 did not differ among themselves on all of the five tests.

## Discussion

This study suggests that the educational format of repetition activity does not influence the long‐term retention of anatomical knowledge at three months after the repetition activities. The 30% loss of knowledge after a period of six months without repetition activities largely agrees with the results reported in a review by Custers (2010). Doomernik et al. (2017), who performed their study with the same curriculum, found a smaller decline in long‐term retention by about 15% after 1.5 years, but in this study, the students had revisited the earlier learned material during the time in between.

This study seems to be the first to investigate the effect of different repetition methods on the long‐term retention of anatomical knowledge in a running curriculum. A few papers examine interventions that decelerate long‐term knowledge loss in running curricula, but these papers differ in at least one very essential aspect from the present study and can therefore not be compared to the present study: Feigin et al. (2007) showed an increase in retention (about 15%) that was due to repeated testing, but their study population revisited the previously learned material during a clinical elective. Logan et al. (2011) showed an increase in retention because of testing, but the retention test was taken only one week after the original learning phase. Finally, Jurjus et al. (2015) showed an increase of 14% in anatomical knowledge immediately after repetition activities, but they did not measure long‐term retention.

Different factors may have contributed to the finding that students who revisited previously learned knowledge with passive and active learning methods scored similarly on both the post‐repetition test and the long‐term retention test. One factor, which is also a limitation of this study, is that the students’ participation was voluntary. Students were aware of being a research subject and may have adjusted their behavior accordingly. Students attending the traditional lecture, representing the passive learning method in this study, may have paid “above average” attention to the lecturer. They may have strained themselves to remember the information more than they normally would during an ordinary lecture. This increased engagement may have changed the lecture from a passive to a more active learning method, possibly increasing the resulting test scores of these students.

The repetition methods chosen for this study are methods that were already integrated into the curriculum at Nijmegen at the time of this study. Other possible activities, for example, game‐based (Rondon et al., 2013) or drawing‐based (Balemans et al., 2016) activities, were not considered for that reason.

There are also a few studies that compared different repetition methods and/or retrieval strategies. Blunt and Karpicke (2014) showed that both writing down and using concept mapping to retrieve earlier learned material produced better performance than did additional learning, but these two methods do not differ from one another. Bae et al. (2019) compared the taking of multiple‐choice tests, test generation, and free recall as methods for retrieving knowledge. They showed that test‐taking and free recall were the most effective in knowledge retrieval. However, the latter two studies, again, took only one week between initial learning and retrieval practice. Thus, the data of the present study substantiate the conclusion that in the long run a lecture that repeats previously learned knowledge is just as good as an e‐learning module, a small group learning session, or a multiple‐choice test in facilitating students’ retrieval of knowledge.

Another explanation may be found in how the retrieval of information during the repetition classes was evoked. Retrieval is enhanced in the same way as encoding by factors such as arousal and attention (van Kesteren et al., 2012). It seems to be unknown what a necessary degree of attention should be for the retrieval of information from schemas in the brain. In this study, a lot of attention and arousal cues were offered to the students. Therefore, it seems that students had more than enough opportunities to address their memory and to retrieve the previously learned knowledge: a lecture in which a teacher recalls it for them; an e‐learning module that incorporates provided study material and specific questions; and a small group session where the studied material, specific assignments, and explanations given by other students that may awaken a previously forgotten term or anatomical image. In addition, this study shows that the amount of long‐term retention is independent of the retrieval method. Therefore, passive learning methods and the taking of tests (see below) may be “good enough” to repeat previously learned knowledge since it stimulates enough retrieval of this knowledge for the students to perform well in subsequent tests.

This study was set up to explore the effect of different repeating activities using tests to measure the effect of these repeating methods. To establish the loss or gain of knowledge during this study, multiple‐choice tests were filled in by the students. Using tests as a measurement method, the testing effect was introduced. Revisiting the same material after 14 weeks by means of a knowledge test enabled the students to recall the same amount of knowledge after an additional 14 weeks, compared with the other learning methods. Roediger and Karpicke (2006) established that the benefits of re‐study through taking a test on the previously learned material could be measured after one week. The present study adds to their research with the finding that a test to recall earlier learned material after a much longer period of 14 weeks is still an efficient method to recall the same initially learned material in the long term.

Many more studies examine the testing effect (e.g., Karpicke and Roediger, 2008; Larsen et al., 2008; Karpicke et al., 2014; Binks, 2018; Larsen, 2018), and all substantiate the effect of testing. The present study shows that the testing effect is as efficient as the retaking of classes to build up stronger schemas and hence retrieval possibilities. It would be compelling to study what a minimal revisiting activity would be that would still enable a student to retrieve a significant amount of knowledge from their long‐term memory. For example, if the mere mentioning by a teacher of where in the past a student has encountered a subject would stimulate a large recall of that subject, then all teachers should be trained to implement this in their educational activities. Besides the mentioning of already learned subjects, showing a figure, a picture, or a mechanism could potentially also be enough for a substantial recall of earlier learned knowledge.

### Limitations of the Study

The first limitation is the already‐mentioned extra motivation of the students, that is, knowing that they were enrolled in a study and would be tested. It was argued in a previous section of this article that this motivation may have changed the lecture from a passive to a more active repetition activity. Furthermore, the students in the other groups may also have strained themselves to excel in the tests. If that has been the case, one would expect that the students in the more active learning method groups, being the method that yields a higher learning gain (see introduction), would have shown higher results than the other group. On the other hand, if the levels of straining would have been exceptionally high for all groups, a ceiling effect might have been reached, meaning that no matter which repetition method would have been applied, the learning gain would have stabilized at the same high level for all groups.

Another issue is the assumption that the groups were similar in their important characteristics, such as prior knowledge, study motivation, and learning abilities. Although the groups were constituted from a rather homogeneous population—all of about the same age, almost all fresh out of high school, all selected by cognitive and psychological tests for medical school, and almost all with above average high school grades—specific demographics (except for gender and age), psychological characteristics, and educational characteristics were not collected or controlled for. It is reasonable to assume that in the studied curricula less than 5% of the students possess a different educational background, having completed most likely one or a few years of study in biology. The effect of this on the results is judged as minimal, particularly given the similarity in the original test scores for all groups.

The composition of Group 1 is another major concern. This group was not tested during the post‐course test. It is not that they did avoid the post‐course test, but they decided not to attend the voluntary classes at the time that the post‐course tests were offered during these classes without advance notice. Due to their absence, it could be deduced that these students may have had a different, presumably lower, study motivation, while the students that were present for the voluntary classes may have been high achievers. Nevertheless, there are no substantial reasons to infer that Group 1 would have shown a different knowledge level at that time since the post‐course test was one day before the summative examination of the first‐year course for all students.

Ideally, several additional groups would have been added to this study, for example, a group comprised of students who did repetition activities during the return day without being tested. These additions would have helped to pinpoint whether the long‐term retention of Groups 3, 4, and 5 was an effect of the repetition activity itself or a combined effect of being tested and engaging in a repetition activity (Norman et al., 2002). Group 2 could also have been asked to fill in the post‐repetition test. This change would have made Group 2 differ in one aspect instead of two (no repetition activity and no post‐repetition test) from Groups 3, 4, and 5.

Although a drop‐out percentage of 40% was expected from the beginning, the study was originally designed for 30 students to be in every experimental group at the end of the study. This constraint restricted the feasible number of groups to five during the design of this experiment.

Although similarity in the offered images and texts was strictly controlled for, only the students in Group 5 had anatomical preparations at their disposal. This decision does not seem to have had any effect on their learning gain, since they scored about the same in the post‐repetition test as the other groups.

The poor to moderate reliability of the tests is of some concern. The alpha of the pre‐course test (−0.022) reflects a lot of random answers, which is not surprising because there is little to no knowledge to be measured. The alphas of the post‐course and post‐repetition test (0.634 and 0.652, respectively) are much better, but still moderate. In the curriculum of this research, the moderate results for Cronbach’s alpha would not be acceptable for a summative assessment. About 100 questions per multiple‐choice test would be the standard, enhancing reliability significantly. However, this would take much more time to complete, which is the reason to limit the tests in this study to 40 question each. It is expected that students are less likely to volunteer if the duration of the tests would drastically increase.

The construct validity of the tests was good. The same set of objectives was used to design the repetition material and to construct the tests, ensuring constructive alignment (Biggs, 1996).

Kendall tau‐B’s correlation coefficient of the long‐term retention test in relation to the pre‐course test, the post‐course test, the pre‐repetition test, and the post‐repetition test (0.133, 0.431, 0.329 and 0.453, respectively) reflected an increasing concordance between tests when the knowledge of the students increases. Nevertheless, there was no external criterion available to assess the validity of the tests in this study. The validity argument (Messick, 1998) of the tests that were used in this study is built from (1) the scores on the subsequent tests and (2) their association. The participants had better scores after learning and after repeating, in respectively the post‐course and post‐repetition test, compared to the pre‐course and post‐repetition test. In other words, where the participants were expected to have better knowledge/competence (after learning/repeating) they scored higher. This is an indication of convergent validity.

## Conclusion

This study examined the long‐term retention of anatomical knowledge after the engagement in various repetition activities. Anatomical knowledge was assessed in a real‐world educational setting and using a realistic time frame for potential knowledge loss typical of medical curricula. The results suggest that the method applied during repetition activities, whether active or passive, does not influence the long‐term retention of the rehearsed knowledge. Although the final number of students in the repetition groups were disappointingly small, statistics show good levels of significance to substantiate the findings. On top of this, the study also underpins that any rehearsing act, including an educational test, is as beneficial as a scheduled repetition activity for the long‐term recall of that knowledge by students. So, starting a subsequent course or clinical rotation with repetition is very advantageous when the intention is to build upon or apply previously learned knowledge. The format of the repetition could be a multiple‐choice test, a traditional lecture in a massive hall, collaborative small group work, or an e‐learning module. If durable education in a spiral curriculum is strived for, teachers should be aware to facilitate the retrieval of earlier learned knowledge at the right position in the curriculum or at the right time during the students’ individual learning path.

## Notes on Contributors

JAN G.M. KOOLOOS, Ph.D., is an associate professor and a senior lecturer in the Department of Anatomy at the Radboud University Nijmegen Medical Center, Nijmegen, The Netherlands. He teaches gross anatomy, embryology, histology, and functional anatomy of the locomotor system in a variety of curricula. He is also the Co‐President of Faculty Development and involved in several research projects on anatomy education.

ESTHER M. BERGMAN, Ph.D., is a research coordinator within the Zuyderland Academy of the Zuyderland Medical Center in Sittard‐Geleen and Heerlen, The Netherlands. She stimulates and facilitates scientific research performed by all health care professionals of Zuyderland Medical Center. Previously, she worked as an assistant professor of anatomy at the Radboud University Medical Center in Nijmegen, The Netherlands.

MARIEKE A.G.P. SCHEFFERS, M.Sc., graduated with honors in the field of educational sciences from the Radboud University Nijmegen, The Netherlands. She currently works as an educational scientist, specialized in developing training programs, (digital) learning materials, and examinations for educational programs in various fields of secondary vocational education.

ANNELIEKE N. SCHEPENS‐FRANKE, Ph.D., is an assistant professor and a lecturer in the Department of Anatomy at the Radboud University Nijmegen Medical Center, Nijmegen, The Netherlands. She teaches anatomy and histology, and focuses on embryology and anatomy of the urogenital system. In addition, she coordinates the plastination activities at the department.

MARC A.T.M. VORSTENBOSCH, Ph.D., is an associate professor and a lecturer in the Department of Anatomy of Radboud University Nijmegen Medical Center, Nijmegen, The Netherlands. He teaches gross anatomy, functional anatomy of the locomotor system, and head and neck anatomy. As a researcher, he is involved in several research projects on anatomy education and the assessment of medical competence.
