# Supplementary material for: The Effect of Passive and Active Education Methods Applied in Repetition Activities on the Retention of Anatomical Knowledge
Source: Anat Sci Educ. 2019 Nov 6;13(4):458–66. doi: 10.1002/ase.1924 (PMC7383800; doi:10.1002/ase.1924)
Supplement: Supplementary file 1 [file ASE-13-458-s001.docx]

**Supplementary Material Appendix:**

Example of seven from the 40 multiple choice questions that were used in this study.

The ligamentum inguinale runs at the lower border of the inguinal canal. This ligament is made of the aponeurosis of one of the abdominal muscles. Which muscle is this?

1. m. obliquus abdominis externus
2. m. obliquus abdominis internus
3. m. transversus abdominis

The ureters run from the kidneys to the bladder. Where do the ureters enter the bladder?

1. above and behind
2. above and in front
3. below and behind
4. below and in front

Which of the following organs emerges as a fusion in the mid-line of a left and right tube?

1. bladder
2. tuba uterina
3. uterus

Which of the structures a) to d) develops from the intermediate mesoderm?

1. colon ascendens
2. kidney
3. peritoneum
4. spinal cord

The diaphragm consists of a centrum tendineum and a muscular part. Which structure runs through the centrum tendineum?

1. aorta
2. oesophagus
3. vena cava inferior

The os coxae is constituted from three parts. Where do these three parts come together?

1. acetabulum
2. promontorium
3. symphysis
4. crista iliaca

Every rib shows two joints with the vertebral column. These joints are located on

1. the processus spinosus and the vertebral body
2. the processus spinosus and the processus transversus
3. the vertebral body and the processus transversus
